# Exploring the Complex and Multifaceted Interplay between Melanoma Cells and the Tumor Microenvironment

**DOI:** 10.3390/ijms241814403

**Published:** 2023-09-21

**Authors:** Magdalena Kuras

**Affiliations:** 1 Department of Biomedical Engineering, Lund University, 221 00 Lund, Sweden; magdalena.kuras@bme.lth.se; 2 Section for Clinical Chemistry, Department of Translational Medicine, Lund University, 205 02 Malmö, Sweden

**Keywords:** malignant melanoma, tumor microenvironment, metabolic reprogramming, phenotype switching, cellular plasticity, molecular classifications, mutational burden, treatment resistance

## Abstract

Malignant melanoma is a very aggressive skin cancer, characterized by a heterogeneous nature and high metastatic potential. The incidence of melanoma is continuously increasing worldwide, and it is one of the most common cancers in young adults. In the past twenty years, our understanding of melanoma biology has increased profoundly, and disease management for patients with disseminated disease has improved due to the emergence of immunotherapy and targeted therapy. However, a significant fraction of patients relapse or do not respond adequately to treatment. This can partly be explained by the complex signaling between the tumor and its microenvironment, giving rise to melanoma phenotypes with different patterns of disease progression. This review focuses on the key aspects and complex relationship between pathogenesis, genetic abnormalities, tumor microenvironment, cellular plasticity, and metabolic reprogramming in melanoma. By acquiring a deeper understanding of the multifaceted features of melanomagenesis, we can reach a point of more individualized and patient-centered disease management and reduced costs of ineffective treatments.

## 1. Introduction

Melanoma, a word derived from the Greek melas “dark” and oma “tumor”, emerges from melanocytes, a type of specialized dendritic cells of neural crest origin. Melanocytes can be found in the epidermis of the skin, along the choroidal layer of the eye, on mucosal surfaces, and in the meninges [1]. Cutaneous melanoma has a heterogeneous nature and a strong propensity to metastasize to other organs [2,3]. The continuous increase in melanoma prevalence is becoming a major clinical problem and will be associated with even higher treatment costs in the coming years. According to WHO (2020) and GLOBOCAN (2020), melanoma incidence and mortality due to melanoma are the highest in Australia and New Zealand, followed by North America and Northern and Western Europe. An average yearly increase of more than four percent has occurred globally for the past twenty years [4]. Intermittent sun exposure (characterized by a history of sunburns along with a weakened immune system), a family history, and prior removal of melanomas are considered significant risk factors [3,5,6]. Melanoma accounts for about 1–4% of all skin cancers globally. Although it is less frequent, it is much deadlier than other skin tumors [6]. In early-stage melanoma, surgical removal of the tumor has a curable outcome in around 90% of the patients. However, for patients with disseminated disease, the outcomes are still poor, despite the past decade’s emergence of immune checkpoint inhibitors and targeted treatments. Here, recent advances in melanoma biology are reviewed, including the interactions between melanoma cells and the tumor microenvironment. By recognizing the complex relationship between melanomagenesis, cellular plasticity, metabolic reprogramming, and mutations, we can reach a point of more individualized and patient-centered disease management.

## 2. Melanocyte Function and the Emergence of Melanoma

**Figure 1:**
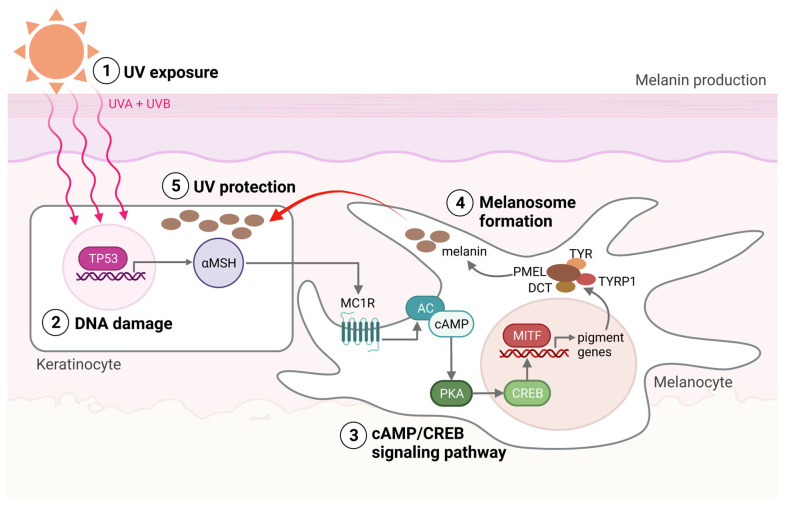
Melanin production. (1) The production of melanin, a process called melanogenesis, is activated upon UV exposure. (2) UV radiation damages the DNA in the keratinocytes, activating the TP53 pathway, which results in the production of αMSH. αMSH is secreted from the keratinocytes and binds to the MC1R on the melanocyte. (3) cAMP levels are then increased within the melanocytes, activating protein kinase A (PKA). PKA activation induces the recruitment of CRE-binding (CREB) protein and thereby the transcriptional activity of MITF. (4) MITF activates the transcription of pigment genes, including *TYR*, *TYRP1*, *DCT*, and *PMEL*, which are transported to the membrane-bound melanosome. (5) Matured melanosomes are then transferred from melanocytes to keratinocytes to protect them against UV light. Created with BioRender.com (accessed on 19 September 2023).

Within the melanocytes are melanosomes that produce the pigment melanin [7]. In response to UV-induced DNA base damage, keratinocytes in the skin produce a melanocyte-stimulating hormone (αMSH) that binds to the melanocortin receptor 1 (MC1R) on melanocytes, ultimately leading to the activation of microphthalmia-associated transcription factor (MITF) (Figure 1). MITF upregulates the expression of enzymes required for melanogenesis and is considered the main regulator of melanocyte differentiation. Amplification in MITF is present in about 20% of melanomas and is associated with reduced five-year survival [7]. Melanin and the distribution of melanosomes in the epidermis are among the most critical factors in protecting human skin from the harmful effects of UV radiation [8,9]. UVB (280–315 nm) radiation is considered a primary mutagen because it is absorbed directly by DNA, inducing DNA base damage. On the other hand, UVA (315–400 nm) is mainly responsible for indirect DNA damage by generating reactive oxygen species (ROS) [10,11]. The damage to the DNA can cause mutations in genes involved in melanoma development and is thought to be the first step toward disease formation.

## 3. Melanoma Diagnosis

The preferred method to diagnose melanoma is surgical excision, with appropriate margins, followed by a histological examination of the skin lesion. Parameters such as histological subtype, number of mitoses, and Breslow thickness are determined, as well as the presence or absence of ulceration and microsatellites. Additional information regarding the growth phase, the presence or absence of tumor-infiltrating lymphocytes (TILs), signs of regression, and vascular or perineural involvement may also be assessed. If there are uncertainties regarding the diagnosis, immunohistochemical analysis provides tools to differentiate malignant lesions from benign nevi. The melanoma markers MLANA (MART-1), TYR, HMB45 (PMEL), SOX10, S100, and the proliferation marker MIB-1, which recognizes the Ki67 antigen, are used to diagnose primary and metastatic melanoma. Using these markers, melanoma can be distinguished from other cancer types such as epithelial tumors, neuroendocrine tumors, sarcomas, lymphomas, and germ cell tumors [12,13,14,15,16,17,18]. However, some degree of difficulty remains in distinguishing certain melanoma histotypes from other skin lesions [19]. The use of SOX-10 has solved many cases, but misdiagnosis remains, which can have detrimental effects on the patient [19,20,21]. To further aid the diagnostic process, Preferentially Expressed Antigen in Melanoma (PRAME) [22] has been proposed as a complementary immunohistochemical diagnostic marker [23]. PRAME is a cancer-testis antigen expressed in a variety of tumors and plays a central role in the development of germ cells and the pathogenesis of testicular germ cell tumors [24,25]. In comparison to immunostaining for MLANA and Sox10, PRAME stains normal melanocytes to a much lesser extent. If inflammation, cauterization artifacts, or melanocyte hyperplasia is present, PRAME may offer a sharper image of where a melanoma lesion ends [26]. However, the expression of PRAME in nevi, solar lentigines, and benign skin cancer may represent diagnostic pitfalls and needs to be researched further.

## 4. Melanoma Susceptibility

About 10% of melanomas occur in patients with a family history of melanoma [27,28]. Although most genetic alterations associated with melanoma development are somatic, the underlying presence of heritable melanoma risk genes is essential to disease occurrence. This is believed to be the case even in “sporadic” melanoma [29,30]. In the familial atypical multiple mole-melanoma (FAMMM) syndromes and the melanoma–astrocytoma syndrome (MAS), germline mutations in *CDKN2A* and *CDK4* are the most frequent genetic abnormalities [28]. *CDKN2A* encodes two crucial tumor suppressor proteins, p16^INK4A^, and p14^ARF^, which, together with *CDK4*, function as cell cycle regulators [31,32]. Germline mutations in *CDKN2A* and *CDK4* or in *BAP1*, *TERT*, *POT1*, *ACD*, and *TERF2IP* are considered predisposition mutations with high penetrance [3,30]. Medium and low penetrance genes such as *MITF* and *MC1R* are more prevalent in the population, but alone, they are unlikely to result in melanoma development. However, a combination of mutations in several of these genes may be enough to promote disease development.

Polymorphism of the *MC1R* gene has been found to play a significant role in sporadic melanoma of the skin. *MC1R*, the key regulator of skin pigmentation, has more than 200 coding region variants identified, many within the European population [33]. Polymorphisms in the *MC1R* gene give rise to diverse skin pigmentation phenotypes, among which red hair, freckles, and fair skin express low pigmentation. Increased vulnerability to melanoma is a consequence of a quantitative shift of melanin synthesis from eumelanin to pheomelanin [33]. Furthermore, specific variants of *MC1R* are believed to increase the penetrance of *CDKN2A* mutations, doubling the risk of melanoma compared to a *CDKN2A* mutation alone. Likewise, the coexistence of certain *MC1R* variants and a somatic *BRAF V600E* mutation contributes to increased tumor growth [34,35]. An interaction between *MC1R* and the tumor suppressor *PTEN* has also been proposed, where wild-type *MC1R*, but not the variants, associates with *PTEN* and protects it from degradation, thereby stimulating an immune response [36].

## 5. Dysregulated Signaling Pathways in Melanoma

**Figure 2:**
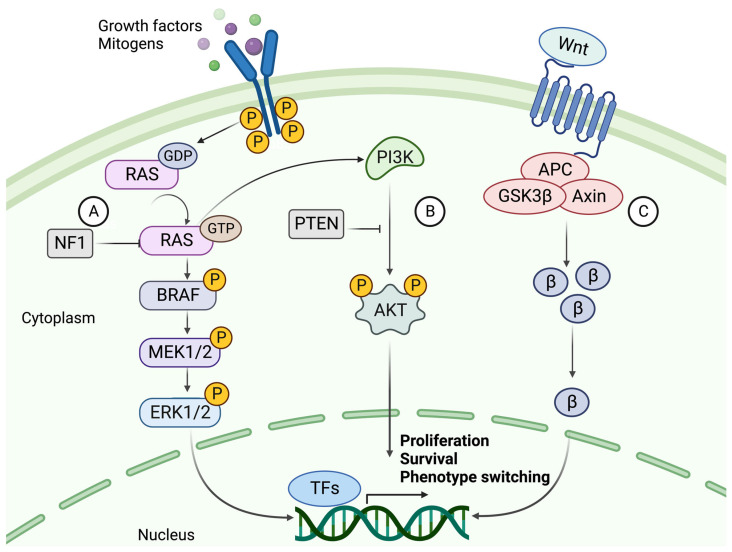
The signaling cascades of MAPK, PI3K, and canonical WNT pathways. (**A**) The NRAS-BRAF MAPK pathway is activated by binding a growth factor (GF) or mitogen to an RTK. Upon activation, the RAS protein phosphorylates (P) MEK1/2, phosphorylating ERK1/2. ERK can then translocate to the nucleus and activate transcription factors which promote proliferation and progression through the cell cycle. NF1 hampers this cell cycle progression by converting RAS to its inactive GDP-bound form. (**B**) The activation of PI3K signaling by GTP-bound RAS. PI3K activates AKT through phosphorylation using a second messenger (PIP3 not shown). AKT is a kinase that mediates the phosphorylation of protein substrates, subsequently affecting the cell cycle and survival of the tumor cell. PTEN acts as a suppressor of this pathway by dephosphorylating PIP3, thereby blocking the activation of AKT. (**C**) The canonical WNT signaling pathway and its main effector, β-catenin (β). β-Catenin is activated upon binding a WNT ligand to the G protein-coupled receptor (GPCR), whereby it is prevented from degradation and instead accumulates in the cytoplasm. β-Catenin then relocates to the nucleus, where it acts as a co-activator of transcription of genes such as those related to EMT-like phenotype switching. Created with BioRender.com (accessed on 19 September 2023).

The mutation rate in melanoma tumors, measured as the number of mutations per Mb, exceeds that of all other cancers according to The Cancer Genome Atlas (TCGA) data [2,37]. The high rate of somatic mutations makes it difficult to distinguish between causative and bystander mutations. The major players involved in melanoma formation include cell-autonomous mutations of the MAPK, PI3K, and WNT signaling pathways (Figure 2) [5,38].

### 5.1. The Mitogen-Activated Protein Kinase (MAPK) Pathway

The most common mutations in melanoma involve the MAPK signaling pathway, where acquired mutations in the genes encoding two kinases, BRAF and NRAS, result in continuous activation and aberrant cell proliferation. Constant activation of MAPK signaling leads to increased expression of cyclin D1, which interacts with CDK4/6 to promote phosphorylation and inhibition of the retinoblastoma (Rb) family of transcriptional repressors, enhancing E2F-dependent transcription of S-phase genes, facilitating G1-S phase transition and consequently cell cycle progression [39]. Upon acquirement of a *BRAF* mutation, a benign melanocytic lesion does not switch to malignancy. Instead, it enters a state of oncogene-induced senescence (OIS), leading to cell-cycle arrest [3,40]. OIS results in tumor suppression by blocking cell proliferation and facilitating the recruitment of immune cells to eliminate the damaged melanocytes [41].

*BRAF* mutations are found in about half of all melanocytic lesions and are thought to occur early in the disease. Most mutations occur at the *V600* position, where a valine-to-glutamate substitution (*V600E*) is the most common (~80–90%). *V600K*, *V600D*, and *V600R* account for another 10% to 15% of mutations. *BRAF* mutations are more frequent in melanomas that develop in sun-exposed skin. The mutated BRAF kinase can activate its downstream effector MEK, independent of RAS activation. MEK then activates ERK, which relocates to the nucleus and alters gene transcription by increasing the expression of various transcription factors, such as c-MYC and MITF (Figure 2) [42,43]. MITF coordinates many different signaling pathways in melanoma, including cell-cycle regulation, differentiation, and migration, depending on its expression levels [44]. Generally, low MITF activity promotes invasion and cell cycle arrest, while high activity favors proliferation and differentiation [45,46].

*NRAS* and *BRAF* mutations are almost always mutually exclusive, where *NRAS* mutations occur in about 20% of melanoma cases. In addition to MAPK pathway activation, oncogenic RAS acts as a positive upstream regulator of the PI3K pathway [47]. Thus, activation of RAS transcription leads to downstream activation of two interconnected pathways. While both cause cell proliferation, dissemination, and survival of tumor cells, the PI3K-AKT signaling pathway contributes more strongly to apoptosis prevention, whereas the RAF-MEK-ERK pathway is more active in proliferation and invasion [48,49]. Patients with NRAS mutations, often in the Q61 position, tend to have a worse prognosis and shorter median survival due to the aggressive nature and lack of treatment for this mutation [50].

### 5.2. The PI3K/AKT Signaling Pathway

To overcome OIS in a *BRAF*-mutated melanocytic lesion, subsequent mutations of the PI3K pathway are frequently acquired [40]. The coexistence of mutations in these pathways has been shown to overcome *BRAFV600E* OIS by the loss of PTEN expression or by overexpression of AKT3, a downstream target of PI3K (Figure 2). PTEN-negative or AKT3-overexpressing melanomas do not undergo apoptosis in response to BRAF inhibition [51]. PTEN is also thought to inhibit MAPK signaling by decreasing the phosphorylation of MEK and ERK [52]. Interestingly, *BRAF* mutations were found in both the nevus and melanoma parts in melanoma biopsies, while activation of the PI3K pathway was detected in the melanoma portions only [36,53]. This indicates that the PI3K/AKT pathway is activated during the progression to malignant melanoma, most likely to overcome OIS.

### 5.3. Canonical and Noncanonical WNT Signaling

Normal WNT signaling is required for melanocyte development, while aberrant WNT signaling is known to contribute to melanoma formation [38]. The canonical WNT pathway mainly contributes to the development of primary melanoma by suppressing p16INK4A, overcoming OIS, and increasing proliferation. By contrast, the noncanonical pathway is primarily involved in metastasis formation by disrupting cell polarity and increasing migration capabilities [44]. Constitutively activated canonical WNT signaling via β-catenin has been shown to act synergistically with the MAPK pathway, by suppressing the expression of p16INK4A and cooperating with NRAS in the transformation of a melanocytic lesion into melanoma. In a melanoma mouse model with a BRAFV600E mutation and inactivated PTEN, β-catenin acted as the central mediator of metastasis development and a regulator of both the MAPK and PI3K pathways [54]. Additionally, WNT signaling is known to be one of the key regulators of epithelial-mesenchymal transition (EMT), and it plays an important role in the EMT-like phenotype switching that occurs during melanoma progression [55].

### 5.4. The Role of KIT, NF1, TERT, and TP53 in Melanoma

Other essential effector molecules in melanoma formation include KIT, NF1, TERT, and p53. *KIT* is a proto-oncogene that encodes a receptor tyrosine kinase (RTK) and is considered a driver mutation in melanoma. It is found to be mutated in about 1–3% of melanoma tumors [56]. KIT activates the MAPK and PI3K pathways leading to cell proliferation and survival. *NF1* is a tumor suppressor that negatively regulates the MAPK and PI3K pathways. *NF1* mutations are prevalent in melanomas that are wild-type for *BRAF* and *NRAS,* NF1 are considered driver mutations in these patients [57]. Mutations in the *TERT* gene increase transcription from the TERT promoter, thereby preventing cancer cells from undergoing apoptosis [58]. Mutations in the promoter region of *TERT* are considered driver mutations because of their association with familial melanoma and high frequency (70–80%) in sporadic melanoma. Mutations in the well-known tumor suppressor *TP53* are also present within melanoma tumors but less frequently than in other cancers. Point mutations are found in about 10–20% of melanomas, suggesting that these tumors use different mechanisms to evade tumor suppression by the p53 protein [36,57].

## 6. The Tumor Microenvironment

**Figure 3:**
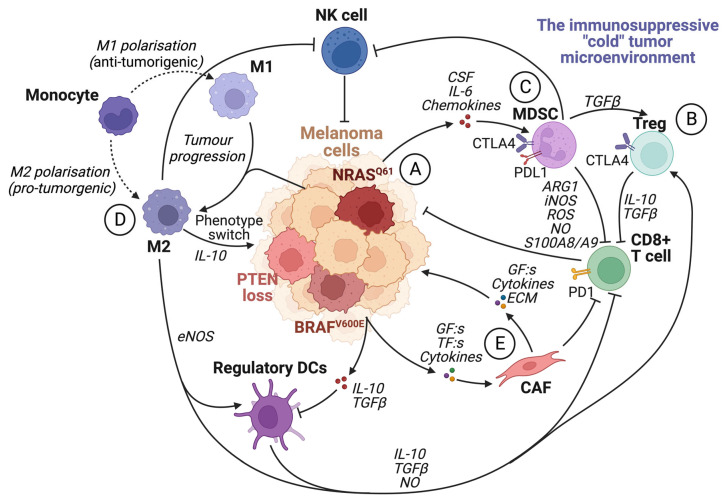
The immunosuppressive tumor microenvironment. The recruitment of various cells modulates the TME by the secretion of cytokines and chemokines by tumor cells and other infiltrating cells. Most cells within an immunosuppressive tumor microenvironment inhibit the activation and function of cytotoxic CD8+ T cells. (**A**) Tumor cells can induce the activity of Treg cells, tumor-associated macrophages (M2), and MDSCs by secreting growth factors such as VEGF. They also facilitate the transformation of fibroblasts into cancer-associated fibroblasts and enhance the expression of PD1 on CD8+ T cells. (**B**) Treg cells inhibit CD8+ T cells and NK cells by upregulating CTLA4 and releasing IL-10 and TGFβ. (**C**) MDSC expression of PDL1 inhibits T-cell activation by binding to PD1. Furthermore, MDSCs promote Treg cell proliferation in a TGFβ-dependent manner, boost angiogenesis in the tumor microenvironment, and contribute to the phenotypic switch in melanoma cells. In addition, MDSCs hinder CD8+ T cells by releasing arginase I and S100A8/A9 and metabolites such as ROS, NO, and iNOS. (**D**) TAMs promote regulatory DC maturation, inhibition of CD8+ T cells and NK cells, and facilitate phenotype switching in melanoma cells by IL-10 signaling. (**E**) Cancer-associated fibroblasts (CAFs) can induce immunosuppression by inhibiting CD8+ T cells. CAFs also secrete TGFβ, CXCL12, matrix metalloproteinase 2 (MMP2), and IL-6, which promote tumor proliferation and invasion. Created with BioRender.com (accessed on 19 September 2023).

The tumor microenvironment (TME) is known to have a central role in cancer development. A pro-tumorigenic microenvironment is essential for a tumor’s survival and progression [59]. Compared to the microenvironment surrounding normal tissue, the TME differs in its extracellular matrix (ECM) composition, metabolism, nutritional status, pH, and oxygen levels [60]. The TME consists of a complex mixture of fibroblasts, immune cells, and endothelial cells embedded in the ECM. There is continuous reciprocal communication between tumor cells and the microenvironment, where tumor cells secrete stimulatory growth factors, chemokines, and cytokines, as well as induce changes in glucose and oxygen levels [38,59,60]. This results in the recruitment of stromal cells, immune cells, and vascular cells, which remodels the surroundings and creates a pro-tumorigenic microenvironment (Figure 3). The ability of the tumor microenvironment to drive melanoma progression, invasion, and metastasis involves increased cell proliferation and survival of the tumor cells, metabolic reprogramming, and altered cellular plasticity where the cancer cells acquire stem-like properties and become more neural crest-like [61].

### 6.1. Metabolic Reprogramming of the Tumor Microenvironment

Oncogenic reprogramming of cellular metabolism is an essential feature of melanoma progression. It is triggered by genetic alterations and adaptations, forming a microenvironment that lacks nutrients and oxygen [60,62,63]. Metabolic reprogramming from oxidative phosphorylation (OXPHOS) to a glycolytic phenotype has long been considered a hallmark of cancer [64]. Indeed, a key feature of *BRAFV600E* mutated melanoma is the metabolic reprogramming from mitochondrial respiration to glycolysis [63]. However, there is increasing evidence linking mitochondrial pathways to cancer development and progression [65,66,67,68]. Mitochondrial metabolism impacts tumor development by increasing ROS as a byproduct of OXPHOS, supporting the genomic instability required for transforming a melanocytic lesion into a melanoma tumor [63]. ROS also triggers oncogenic signaling of the MAPK pathway [66]. It is becoming evident that melanoma cells are dependent on mitochondria after inhibition of oncogenic MAPK signaling, linking mitochondrial dynamics, oncogenic MAPK signaling, and metabolic reprogramming to tumorigenesis. An association between the metabolic state of a melanoma tumor and the response to immunotherapy has been observed since higher expression levels of mitochondrial genes correspond with better response to immune checkpoint inhibitors [69].

Additionally, tumor cells secrete growth factors, transcription factors, and cytokines to modify the microenvironment by reprogramming fibroblasts to cancer-associated fibroblasts (CAFs) (Figure 3). Although CAFs can originate from epithelial cells, endothelial cells, HSCs, and CSCs, among others, fibroblasts represent the largest population of stromal cells within the TME [70]. In the early stages of tumorigenesis, activated fibroblasts act as tumor suppressors which eventually develop into CAFs as the disease progresses. CAFs are characterized by the expression of α-smooth muscle actin, vimentin, desmin, and fibroblast-activation protein (FAP) [59,71]. These fibroblasts supply the tumor with growth factors, cytokines, and metabolites and stimulate blood vessel formation. CAFs rely upon aerobic glycolysis, a characteristic of highly proliferating cells, which promotes the metabolic adaptation of the progressing tumor. The tumor stroma can impact the aggressiveness of cancer cells not only through signaling but also through mechanical pressure and tissue stiffness, mainly stimulated by the LOX proteins [72,73]. A high frequency of CAFs and ECM proteins in the stroma forms a physical barrier surrounding the tumor. This barrier increases the interstitial pressure and hypoxia within the tumor, partly explaining why a tumor with a high stromal content often is associated with an inadequate response to therapy and a poor outcome [74,75]. In response to the low oxygen levels, the cancer cells upregulate HIF1α, a transcription factor that controls genes contributing to angiogenesis, migration, metabolism, and metastasis [76].

### 6.2. Phenotype Switching of Melanoma Cells

**Figure 4:**
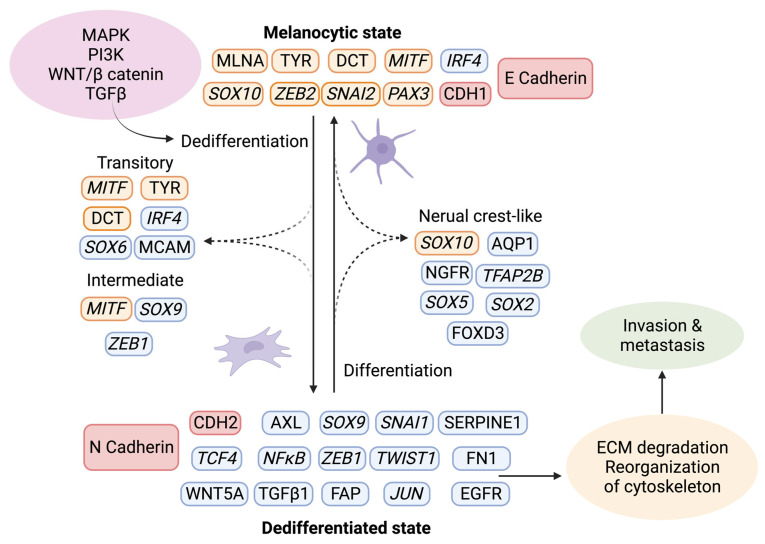
Phenotype switching in melanoma. Upon activation of the signaling pathways MAPK, PI3K, WNT, and TGFβ, a changed expression of specific markers leads to ECM degradation and reorganization of the cytoskeleton, which facilitates invasion and metastasis. Using single-cell approaches, it has become evident that phenotype switching is not binary but rather a multistep process in which cells transit through a series of intermediate cell states expressing combinations of epithelial and mesenchymal phenotypes. These intermediate states can simultaneously display proliferation, invasion, and stemness features. Transcriptional regulators are highlighted in italics. The red indicates the switch between E- and N-cadherin and the responsible genes CDH1 and CDH2. Melanocytic markers are highlighted in orange, and other markers are in blue. Created with BioRender.com (accessed on 19 September 2023).

The tumor stroma further contributes to melanoma progression by initiating a “phenotypic switch”, whereby the melanocytic characteristics are lost and exchanged in favor of a more dedifferentiated phenotype (Figure 4) [77]. The plasticity of melanoma cells shares characteristics of neuroendocrine tumors [78], germ cell cancers [24,79], and epithelial tumors. The dedifferentiated mesenchymal-like melanoma cells almost completely lose their melanocytic features and appear nearly undistinguishable from epithelial tumors undergoing EMT [80,81,82].

Given that melanocytes derive from neural crest cells, which underwent EMT during their development into melanocytes, it is not surprising that phenotype switching mimics this process [83]. The phenotypic transition of melanoma cells includes the acquisition of a spindle-like morphology, upregulation of mesenchymal markers, and release of ECM degrading proteins, such as matrix metalloproteinases (MMPs) which further support invasiveness. An additional downregulation of the epithelial cell surface protein E-cadherin in favor of N-cadherin accompanies the phenotypic switch. As a result, the keratinocytes in the skin lose control over the melanoma cells, which gain properties to grow vertically through the epidermis [84]. A parallel upregulation of TGFβ signaling and the above-described pathways (MAPK, PI3K/AKT3, and Wnt/β-catenin) increase the abundance of transcription factors such as MITF, SOX, Snai1/2, TCF4, Twist1, Zeb1/2, PAX3, and NFκB, further facilitating phenotype switching [85,86,87]. Melanoma is believed to progress and metastasize by alternating between the melanocytic/proliferative and dedifferentiated/invasive states, including intermediate (proliferative and invasive), transitory (proliferative and invasive), and neural crest stem cell-like (NCSC-like) states (invasive) [77,88]. In several studies, the two extreme conditions are characterized by a high expression of MITF and a low expression of the RTK AXL in the melanocytic state, and a low MITF and high AXL expression in the dedifferentiated state. The different phenotypes often coexist in the same tumor, meaning that different subpopulations of melanoma cells can simultaneously contribute to both tumor growth and metastasis [46,88,89,90,91,92].

## 7. The Immune System

The immune system plays a crucial role in the fight against cancer. Advances over the past decade in immunotherapy have led to a long-standing but evident strategy for cancer treatment based on activation of the endogenous immune system for tumor recognition [93,94]. However, the treatment of melanoma is still troublesome, mainly due to the plasticity of melanoma cells, which often gives rise to immune evasion, whereby the tumor cells become invisible to the immune cells and are thus able to escape [60].

Three phases can describe the interplay between melanoma cells and the immune system: elimination, equilibrium, and escape [95]. During the early stages of tumor formation, the neoplastic cells are recognized and eliminated by NK cells and cytotoxic CD8+ T cells, hampering tumor initiation [96]. Eventually, as the tumor acquires additional mutations and epigenetic alterations, subpopulations of cells evade the immune system. During the equilibrium phase, the resistant, “invisible” clones expand. However, the immune system can keep the melanoma cells in check by continuously eliminating the “visible” clones [60]. In the last phase of immune evasion, the best-adapted clones can grow without restraint, causing the disease to progress.

### 7.1. The Roles of the Different Immune Cells in Melanoma Development

Melanoma tumors are considered immunogenic, and often, they have a higher infiltration of immune cells compared to other cancer types. These immune cells can have opposing effects, either initiating or inhibiting the immune response [94]. Macrophages constitute a large pool of immune cells in solid tumors, and other cell types include NK cells, lymphocytes, dendritic cells (DCs), mast cells, and neutrophils [60,94]. Macrophages present within a tumor can have very different functions, which are divided into two main groups: pro-tumorigenic (M2) and antitumorigenic (M1) (Figure 3). The M1 macrophages are often more active in the earlier stages of tumor formation and exert immune activation, apoptosis, and inflammatory functions. On the other hand, the M2 macrophages are often the most dominant in the later disease stages. The M2 macrophages promote tumor growth, angiogenesis, invasiveness, and immune suppression. Macrophages utilizing tumor-suppressive functions are often called tumor-associated macrophages (TAMs). TAMs can switch between M1 and M2, depending on the stimulus from the surrounding tissue [60].

Although macrophages are often the most dominant immune cell type, the presence, localization, and activity of TILs in melanoma tumors are the most important features of successful immune surveillance. TILs recognize antigens presented by the tumor cells and mediate cytotoxicity, thus keeping tumor growth in check. However, the tumor’s ability to uphold an immunosuppressive environment can promote progression by chronic exposure to interferons (IFNs), interleukins (ILs), growth factors, and colony-stimulating factors (CSFs) recruiting suppressive immune cells such as Treg cells, myeloid-derived suppressor cells (MDSCs), and TAMs (Figure 3) [97,98]. Additionally, chronic exposure to IFNs upregulates the inhibitory T-cell receptors CTLA-4, PD-1, TIM-3, TIGIT, BTLA, and LAG-3, enhancing resistance to immune checkpoint inhibitors [94,99]. Treg cells and MDSCs often express high amounts of immune checkpoint molecules such as PD-L1, PDL-2, HVEM, and ITAMs. By continuously expressing these molecules, the CD8+ T cells become exhausted, creating a vicious cycle of immunosuppressive signals, facilitating further disease progression [71]. A high Treg cell-to-CD8+T cell ratio in the TME of melanoma is associated with an unfavorable outcome [100]. The appearance of immunosuppressive cells has a great impact on tumor progression and response to ICI. Targeting these cell populations by inhibiting their recruitment to the TME is an appealing approach that could improve and restore T-cell cytotoxicity.

Tertiary lymphoid structures are highly organized specialized immune aggregates within the tumor consisting of B cells, T cells, and DCs. These assemblies surround high endothelial venules (HEVs), which attract naive B cells and T cells [101]. Although the exact mechanisms active within the TLSs are not entirely understood, the presence of TLSs and B cells within the tumor promotes response to ICI in melanoma patients [101,102].

### 7.2. The Spatial Architecture of the Tumor Immune Microenvironment

The composition and molecular properties of immune cells in the tumor and TME are well-studied [103,104,105,106,107]. More recently, the spatial architecture of these cells has received increased attention to elucidate the varying treatment responses to ICIs [108,109]. Technologies including multiplexed IHC and immunofluorescent (IF) imaging [110,111], mass cytometry [112], and single-cell multiomics [109,113] have been developed for the in-depth study of immune cell phenotypes, their spatial patterns, and their interactions with other cells.

Single marker IHC has long been used to study immune cells in melanoma, mainly focusing on their density and distribution and how that impacts prognosis prediction [114,115,116]. Melanomas containing a high number of CD8+ T cells in the stromal compartment and within the tumor parenchyma are considered “hot”, while “cold” melanomas are characterized by a scarce immune infiltrate [117,118]. The use of PD-L1 expression alone to predict treatment response has shortcomings [119]. However, recent studies have shown that combining PD-L1 cell-type expression with tissue localization can have clinical implications [120]. Despite this, the use of single markers to predict prognosis and treatment response has limitations.

Multiplexed imaging techniques, such as brightfield (BF) multiplexed IHC, can visualize up to eight markers simultaneously by labeling different cell populations on the same section [121]. Immune cell markers including CD3 (T cell activation), CD8 (cytotoxic T cells), CD20 (B cells), CD68 (macrophages), CD163 (M2 macrophages), and CD16 (cytotoxic macrophages and NK cells) can then be combined with PD-L1, SOX10, and Ki67 [110,122]. Thus, simultaneous determination of the location and interaction between immune cell subpopulations and melanoma cells can be accomplished. PD-L1 has been found to be upregulated in tumor cells close to CD8+ T cells, which may result in T-cell exhaustion and diminished cytotoxic activity [123]. TAMs are characterized by extreme plasticity and are involved in many aspects of tumor progression and therapy resistance [124]. The association between CD68+ macrophages and patient survival is somewhat ambiguous, although the location of CD68+ macrophages at the tumor border is indicative of worse patient outcomes [123]. The presence of CD16+ macrophages is favorable for treatment response when using combination therapy with PD-1 and CTLA-4 in patients with metastatic melanoma [125]. Furthermore, it has been shown that the existence of intratumoral CD8+ T cells and concomitantly low CD163+ macrophages increase the likelihood of response to ICI, while the opposite has been associated with shorter progression-free survival [126]. Apart from ICI therapy, a specific preexisting intra- and peritumoral distribution profile of T cells and macrophages is associated with resistance to MAPKi in melanoma [126]. The identification of tumor-immune interactions using spatial proximity analyses might open the possibility of exploring alternative therapeutic strategies to enhance clinical response rates in melanoma.

## 8. Molecular Classification of Melanoma—A Way Forward

In addition to the clinical and pathologic classifications of melanoma [14,127,128,129,130], there is increasing interest in molecular classifications that aim to stratify patients into clinically meaningful subgroups to guide treatment selection, prognosis prediction, and patient outcomes.

In 2006, Hoek et al. outlined a transcriptional taxonomy for melanoma, using cell lines based on gene expression profiling [88]. They proposed three groups with different metastatic potentials. Subclasses A and B were proliferative with weak metastatic potential and displayed an NCSC-like transcriptomic signature, while subclass C was less proliferative but with high metastatic potential. The subtypes were primarily driven by WNT- and TGFβ-like signaling. In 2010, Jönsson et al. proposed a molecular stratification of metastatic melanoma samples based on gene expression profiling. Tumors were divided into four distinct subtypes “high-immune”, “proliferative”, “pigmentation”, and “normal-like”, as reflected by their characteristic gene expression [131]. This classification was further developed by Harbst et al. in 2012, demonstrating that the classification of primary tumors could be performed using the same subclasses. The classification combined the subtypes “high-immune” with “normal-like” and “proliferative” with “pigmentation” to obtain low-grade and high-grade subtypes, which were significantly associated with survival [132]. In a work by TCGA in 2015, melanoma tumors were divided based on transcriptomic analyses [133]. Three clusters emerged from their analysis “immune”, “keratin”, and “MITF-low”. Among the metastatic melanoma samples in the cohort, survival differed significantly with patients from the immune subtype surviving longer, followed by the keratin subtype and MITF-low.

**Figure 5:**
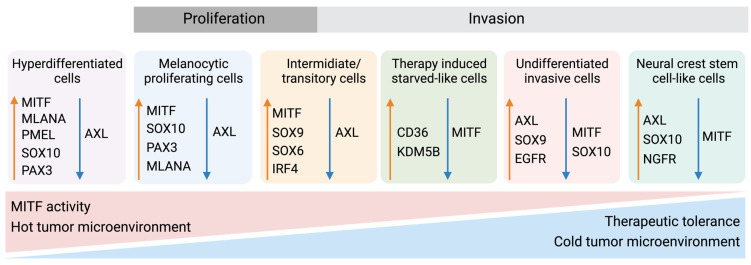
Melanoma phenotype switching and therapeutic tolerance. Up to six differentiation states have been identified in melanoma, each stage being distinguishable by several markers. Stages include a hyperdifferentiated/pigmented state induced by MAPKi, an MITF^high^/AXL^low^ melanocytic stage, an intermediate or transitory stage, a therapy-induced starved-like stage, an MITF^low^/AXL^high^-dedifferentiated stage, and a NCSC-like MITF^low^/NGFR^high^ state [46,81,134,137,138]. Characteristically, the more melanocytic stages comprise a larger proportion of activated immune cells and a “hot” tumor microenvironment—features associated with better therapy response. Conversely, the dedifferentiated and NCSC-like melanomas are absent in immune cells to a greater degree or lack activation of the immune cells. Created with BioRender.com (accessed on 19 September 2023).

Furthermore, Rambow and colleagues utilized a single-cell RNA approach to discover four different melanoma subtypes related to drug resistance [134]. Tsoi et al. investigated the diverse stages of melanoma dedifferentiation and found four distinct subclasses named C1–C4 (Figure 5) [46]. The C1 subtype was considered the most dedifferentiated due to the enrichment of genes related to invasive capabilities such as cell adhesion, motility, and inflammation, similar to observations by Hoek et al. [88] and Konieczkowski et al. [135]. The C2 subtype shared the invasive and inflammatory features of C1 but presented additional characteristics of an NCSC-like phenotype. As expected, both C1 and C2 displayed low levels of MITF and high levels of AXL. C3 was described as an intermediate subtype, with enrichment in NCSC-related genes and genes associated with a more differentiated phenotype. The last subtype, C4, was defined as the melanocytic subtype, with high levels of MITF and other pigment-related genes. The ability of MITF, AXL, and SOX10 to separate the differentiated, intermediate/transitory, NCSC-like, and dedifferentiated subtypes from each other was further demonstrated on the protein level by Lim et al. [136]. Despite different samples and approaches being used in these studies, the different molecular subtypes have similarities. For example, MITF and AXL are recurring and show similarities to the “phenotype switching model” characterized by the ability of melanoma cells to adapt to their microenvironment. Further refinement and validation of these molecular subtypes could be a way to stratify patients and guide treatment planning in a more personalized way.

## 9. The Relationship between Molecular Mechanisms and Treatment Response

The understanding of melanoma pathobiology and treatment of patients with metastatic disease has changed dramatically over the past two decades. A disease that was previously untreatable can now be controlled and even reversed. The most suitable treatment for a patient depends on the disease stage, the melanoma’s location, and the mutational status. Parameters such as age and overall health are also considered [139].

### 9.1. Immunotherapy

Melanoma tumors are considered one of the most immunogenic tumors with a high mutational burden and are therefore well suited for immunotherapy [140,141]. Indeed, melanoma was the first cancer type treated with immune checkpoint therapy [142]. There are currently four approved targets, including CTLA4, PD1, PD-L1, and LAG-3. These proteins are expressed on the surface of T cells, among other cell types, and are involved in signaling pathways that lead to immune suppression. LAG-3 negatively regulates CD4+ T-cell activation and function while enhancing Treg activity and was the most recently approved immune checkpoint target [143]. LAG-3 can act synergistically with PD1 targets [144]. The approved immune checkpoint inhibitors exert their function by binding to these proteins and blocking their activity, thereby reestablishing an antitumorigenic immune environment [145]. In patients with unresectable melanoma, PD1 antagonists, either as monotherapy or in combination with CTLA4, are often considered a first-line treatment, independent of *BRAF* status. However, in some cases, targeted therapy might be more appropriate [140]. In addition to the approved target proteins, novel immune targets including TIM-3, TIGIT, VISTA, and BTLA are being investigated, especially regarding their function in combination with currently approved immune checkpoint inhibitor therapy [95,144]. Despite the major advances of immune checkpoint therapy, nearly half of melanoma patients either do not respond or relapse due to primary or acquired resistance [95]. Intratumoral heterogeneity and cellular plasticity are two important factors of therapy resistance. The MITF^low^/AXL^high^ melanoma phenotypes have been associated with resistance to PD1 inhibitors due to the tumor’s ability to downregulate MHC class I expression and upregulate TGFβ signaling, which recruits CAFs to the tumor site and inhibits NK cell activation and function (Figure 5) [94,146,147,148,149,150,151,152,153].

### 9.2. Targeted Therapy

Approximately 70% of melanoma patients harbor a genetic alteration in one of the main signaling pathways previously described. The MAPK signaling pathway consists of an RTK and the proteins RAS, RAF, MEK, and ERK. Small molecule inhibitors of BRAF and MEK are approved as targets for melanoma therapy (MAPKi) [44]. However, resistance to these inhibitors is an immense problem. When used in combination, increased efficacy combined with reduced toxicity is observed, albeit long-term responses to targeted therapies are rare [145]. In response to MAPKi therapy, melanoma cells often downregulate MITF and upregulate RTKs including AXL, EGFR, and PDGFRβ, resulting in a dedifferentiated MITF^low^/AXL^high^ phenotype [154,155]. This intrinsic resistance has been observed in patient tumors during disease progression on MAPKi therapy [71,156,157,158].

### 9.3. Novel Treatments

New promising therapies to combat melanoma include cancer vaccines based on predicted neoantigens. Due to the high mutation rate in melanoma, the mutational landscape does not overlap between patients. Cancer vaccines induce T-cell reactivity based on the genome of a particular tumor by predicting potential neoantigens [144,159]. However, despite T-cell reactivity being induced, the long-term efficacy still relies on the continuous activation of these cells, which a suppressive TME might hamper. Therefore, combining cancer vaccines with immune checkpoint inhibitors may produce a more efficient therapy response.

Another approach includes nanosystems that aim to improve drug efficacy through personalized and targeted drug delivery. By associating a melanoma treatment, such as immune checkpoint inhibitors or targeted therapies, with a nanoparticle delivery system, the drug can be protected from degradation, and the harmful effects on healthy cells can be minimized [15,160,161]. In both preclinical and clinical studies, intravenous administration is the preferred route for primary and metastatic melanoma models [162].

Adoptive T-cell transfer (ATT) of TILs is another therapy alternative for patients with resistance towards immune checkpoint inhibitors [163,164]. In ATT, ex vivo-expanded TILs are administered to melanoma patients following lymphodepletion with high doses of IL-2 [165]. However, constructing TILs is challenging, and IL-2 is often accompanied by substantial toxicity [140].

The potential synergistic effects of combining CAR T-cell therapy with anti-PD1 and anti-CTLA4 are currently being investigated [144]. In CAR T-cell therapy, the T cells are first removed from a patient, followed by in vitro cultivation and gene manipulation. The cells need to express the CAR antigen receptor, a fusion protein that enables T-cell activation and infiltration of the tumor with subsequent killing of tumor cells. However, CAR T-cell function can be inhibited by a pro-tumorigenic or cold TME with a high presence of immune checkpoint molecules, which could be reversed using immune checkpoint inhibitors.

Therapies aiming to reverse the suppressive signals from the TME are also being investigated [166]. One approach includes TAM-targeting either by reprogramming pro-tumorigenic M2 macrophages into antitumorigenic M1 macrophages, by killing TAMs, or by minimizing their recruitment to the tumor site [60,144]. Additionally, the combination of antiangiogenic modulators and immune checkpoint therapy is also under intense investigation [167]. Other interesting approaches aim to minimize the inhibitory effects of Treg cells, MDSCs, TAMs, and CAFs by inhibiting the signaling between the cells and restoring T and NK cell cytotoxicity. The function and activity of different immune cells are closely connected to the metabolic reprogramming in the TME. The uptake of lipids by dendritic cells reduces their function [168], but it is essential for memory T cells to live for longer periods [169]. The heterogeneous metabolic reprogramming further enhances the complex immune-metabolic network and has important therapeutic implications. Therefore, harnessing metabolic modulators to turn a “cold” into a “hot” TME has great potential to improve the efficacy of immunotherapy and targeted therapies (Figure 5) [68]. These interconnected features should be further investigated, along with phenotype switching and genetic abnormalities, as these phenomena enable melanoma cells to adapt to a variety of stress signals and develop resistance to available therapies.

## 10. Conclusions

Melanoma and many other cancers represent major public health problems. Disease management of melanoma is challenging due to its heterogeneous nature and unpredictable pattern of progression. The emergence of immunotherapy and targeted therapy has prolonged numerous lives, but many patients relapse or do not respond to treatment. Although resistance to immunotherapies may manifest at different times, similar or overlapping mechanisms are often seen [90,91,94]. As outlined, melanoma tumors display a complex landscape with subpopulations of clones with different mutations surrounded by a constantly changing TME. As a result, exceptional intratumoral heterogeneity is observed in both primary tumors and metastases from melanoma patients.

Future studies should investigate how protective microenvironmental signals contribute to OIS in the presence of potent oncogenes. Identification of molecular drivers of the suppressive tumor immune microenvironment may reveal alternative therapeutic strategies. By reprogramming the suppressive TME, immunity can be revived, improving the response to ICI. Studies should also build upon the “phenotype switching model”. Since dedifferentiated phenotypes promote resistance to current treatments, the goal should be to discover new vulnerabilities in these cell states [46,90,134]. Additional promising strategies include targeting multiple drivers of the invasive phenotypes or blocking multiple cell states simultaneously. This is possible due to the identification of markers that define each cell state. A challenge with this approach is the uncertainty regarding which markers drive phenotypic switching and which are bystanders.

The focus must also be shifted toward clinical implementation and translatability of research results [46,134]. The integration of BF multiplexed IHC into clinical practice shows potential, where validation and standardization using image analysis tools could lead to the implementation of these methods in routine diagnostics which would facilitate melanoma diagnosis.

Although progress in understanding melanoma biology has improved significantly over the past two decades, new challenges are emerging, such as the ever-increasing prevalence of melanoma worldwide. We are only beginning to understand how the complex constellation of mutations affects signaling between the tumor and its microenvironment, giving rise to melanoma phenotypes critical to disease progression. As we move forward, our rapidly growing knowledge will hopefully bring melanoma to the level of a chronic, manageable disease.

## Data Availability

No new data were created or analyzed in this study. Data sharing is not applicable to this article.

## Abbreviations

ATT	Adoptive T-cell transfer
CAF	Cancer-associated fibroblast
CTLA4	Cytotoxic T-lymphocyte-associated antigen 4
DC	Dendritic cell
ECM	Extracellular matrix
EMT	Epithelial-mesenchymal transition
GF	Growth factor
HLA	Human leukocyte antigen
ICI	Immune checkpoint inhibitors
IF	Immunofluorescence
IHC	Immunohistochemistry
IL	Interleukin
MAPK	Mitogen-activated protein kinase
MAPKi	Mitogen-activated protein kinase inhibitors
MCR1	Melanocortin receptor 1
MDSC	Myeloid-derived suppressor cells
MHC	Major histocompatibility complex
MITF	Microphthalmia-associated transcription factor
NCSC	Neural crest stem cell
NO	Nitric oxide
OIS	Oncogene-induced senescence
OXPHOS	Oxidative phosphorylation
PD1	Programmed cell death 1
PD-L1	Programmed cell death ligand 1
PI3K	Phosphoinositide 3-kinases
PRAME	Preferentially expressed antigen in melanoma
ROS	Reactive oxygen species
RTK	Receptor tyrosine kinase
TAM	Tumor-associated macrophage
TCGA	The Cancer Genome Atlas Network
TF	Transcription factor
TIL	Tumor-infiltrating lymphocyte
TLS	Tertiary lymphoid structure
TME	Tumor microenvironment
